# Supplementary material for: Infrared thermography applied to lower limb muscles in elite soccer players with functional ankle equinus and non-equinus condition
Source: PeerJ. 2017 May 25;5:e3388. doi: 10.7717/peerj.3388 (PMC5446768; doi:10.7717/peerj.3388)
Supplement: Supplemental Information 2 [file peerj-05-3388-s002.pdf]

Nombre	Altura	Peso	Altura	Altura2	IMC	Retr. Gemelo derecho
Luis Lluch	1,82	81,5	1,82	3,3124	24,6	no
Ruben Valverde	1,68	63	1,68	2,8224	22,32	no
David Rodriguez	1,78	70	1,78	3,1684	22,09	no
Sergio Hervas	1,81	70	1,81	3,2761	21,37	si
Ricardo	1,81	74	1,81	3,2761	22,59	si
Alberto Perez	1,66	69,2	1,66	2,7556	25,11	si
Salvano	1,7	67	1,7	2,89	23,18	si
Cruz	1,78	67	1,78	3,1684	21,15	si
Saralegui	1,89	80	1,89	3,5721	22,4	no
Leguina	1,78	71	1,78	3,1684	22,41	si
Igor	1,83	79	1,83	3,3489	23,59	no
Roberto	1,83	77,4	1,83	3,3489	23,11	si
Galan	1,87	83,5	1,87	3,4969	23,88	no
Marcos	1,86	78	1,86	3,4596	22,55	si
Borrego	1,8	75	1,8	3,24	23,15	si
Valdes	1,77	75	1,77	3,1329	23,94	no
Gorka Unda	1,76	73	1,76	3,0976	23,57	no
Nevado	1,72	70	1,72	2,9584	23,66	si
Gomez	1,85	79	1,85	3,4225	23,08	si
Cristian	1,83	80	1,83	3,3489	23,89	no
Javi	1,85	75	1,85	3,4225	21,91	no

Retr. Gemelo izquierd	Retrac gemelo bil	Retr. Soleo derecho	Retr. Soleo izquierdo
no	no	no	no
no	no	no	no
no	no	no	no
si	si	si	si
si	si	si	si
si	si	si	si
si	si	si	si
si	si	si	si
no	no	no	no
si	si	si	si
no	no	no	no
si	si	si	si
no	no	no	no
si	si	si	si
si	si	si	si
no	no	no	no
no	no	no	no
si	si	si	si
si	si	si	si
no	no	no	no
no	no	no	no

Retrac soleo bil	Temperatura Tibial	Temperatura Tibial	Temperatura tibial anterior m
no	24,3	31,8	30,8
no	26,4	32,5	31,6
no	28	32,5	31,7
si	30	32,7	32
si	29,5	32,3	31,2
si	30	32,7	31,8
si	28,7	31,4	30,4
si	28,3	31,2	30,3
no	26,3	30,4	29,6
si	28,8	32	31,4
no	31	33,1	32,2
si	31,1	33,2	32,4
no	31,8	33,8	33
si	29,9	31,4	30,5
si	29,8	31,6	31,1
no	28,6	32,9	31,8
no	30,6	33,7	33
si	27,7	30,8	30
si	29	30	29,5
no	28,8	32,5	31,7
no	28,2	30	29,2

Temperatura Tibial Anterior minima iz	Temperatura Tibial Anterior maxima iz	Temperatura Tibial Anterior m
22,8	33,2	30,7
28,6	32,2	31,3
28,5	32,2	31,5
29	31,5	30
28,7	31,4	30,7
29,6	31,5	30,7
27,7	30,5	29,4
28,9	31,7	30,7
28,8	30	29,5
30,7	32,7	31,7
30,9	32	31,3
31,1	32,5	31,9
31,5	33,6	33
30,6	31,4	31
29,8	31,4	31
29,4	31,5	30,8
31,4	33,3	32,8
29,9	31,3	30,7
29,5	31	30,4
30	32,6	31,7
27,1	29,6	29

temperatura gemelo izquierd	temperatura gemelo izquierdo maximo a	temperatura gemelo izquierd
27,5	32,4	30,8
27	31,5	30,5
28,5	30,1	29,3
29,4	32,5	30,9
28,9	30,5	29,6
28,6	30,9	30
25,9	29,2	27,7
25,9	31,7	30,6
29,8	31,2	30,6
29,1	31,2	29,8
29,8	30,6	30,3
30,8	31,7	31,4
27,4	31,8	30,3
28,9	30,5	30,1
29,8	31	30,6
29,3	31,2	30,6
30,3	32,3	31,6
29,4	31	30
26,8	29,5	28,3
29,9	31,3	30,3
26,7	28	27,2

temperatura gemelo min	temperatura gemelo maximo derecho a	temperatura gemelo media de
25,9	32,8	30,6
29,5	31,1	30,4
28,5	30,1	29,1
29,7	32,4	31,2
28,5	30,6	29,6
30,1	31,1	30,7
26,7	29	27,8
27,9	31,3	30,3
29,5	31	30,2
28,8	30,2	29,6
29,5	30,7	30,3
30,5	32,2	31,7
29,5	31,8	30,3
29,9	30,7	30,2
29,3	31	30,1
29,4	31	30,5
30,4	32,1	31,4
28,3	30,3	29,4
27,9	29,6	28,5
29,3	32,5	30,7
23,8	30	27,9

temperatura tendon aqu	temperatura tendon aquiles n	temperatura t	temperatura t	temperatura t
16,7	31,2	25,7	19,7	31,5
19,5	28,5	25,4	19,7	30,5
20,7	29,2	25,9	22,1	28,7
22,6	30,6	26,6	21,9	28,5
21,7	28,5	26,2	22,3	28,9
24,3	30,9	28,8	22,5	30,7
21,7	27,8	24,4	22,5	26
22,6	28,3	26,8	23,8	29,3
21,2	26,9	24,3	21,7	27,1
23,5	27,2	25,8	23,5	28,1
23,2	28,2	27	22,1	28,6
24,5	30,2	28,6	23,1	30,9
25,4	31,1	28,7	27	31,5
24,2	29,1	27,6	25,1	31,3
30,2	31,2	30,7	22,3	28,5
24,7	29,6	28,1	24,9	30,1
25,3	31,6	28,1	25,7	31,2
22,5	29,8	26,3	21,4	29,5
21,6	25,1	23,9	22	28,4
20,5	29,6	25,8	22	30
23,4	29,5	26,3	25,4	31,2

temperatura tendc	Temperatura Tibial	Temperatura Tibial	Temperatura tibial anterior m
26,3	28,1	31,5	29,6
25,3	28,4	32,4	32,1
25,6	29	31,5	30,8
27,3	29,6	33,8	32,6
26,5	29,9	32,3	30,9
27,9	28,5	31,6	29,5
25	26,9	29,6	28,3
27,3	27,5	32,2	30,7
23,9	27,2	29,1	28,4
26,1	29,7	32,1	31,2
26,2	27,9	30,1	29,5
29	28,6	30,7	29,5
29,9	26,5	29,3	28,1
28,8	29,9	31,9	30,6
25,2	22,3	26,9	25,1
31,3	28,6	31,9	30,4
28	25,5	31,2	28,8
26,1	29,1	31,7	30,8
24,3	29	31,3	30,1
26	27,4	30,2	28,6
27,8	28,9	31,3	31,3

Temperatura Tibial Anterior minima izq	Temperatura Tibial Anterior maxima iz	Temperatura Tibial Anterior m
26,1	30,8	29,1
29,4	32,5	32,2
26,2	32	31
29,9	34,1	31,9
29,1	31,2	30,4
28,8	29,1	28,7
27,4	30,1	28,7
29,5	32	31
27,5	28,9	28,3
29,2	31,9	30,8
29,8	32	31,5
29,6	31,3	30,6
24,5	29,1	26,7
30,8	32,3	32,3
26,8	29,9	29,1
28,4	31,1	29,9
27,5	30,6	29,2
29,7	32,1	31,2
29,2	32,1	30,9
25,1	30	27,9
28,1	29,9	29,3

temperatura gemelo izquierd	temperatura gemelo izquierdo maximo d	temperatura gemelo izquierd
29,7	31,8	31
30,3	32,3	31,3
28,1	31,5	29,3
29,4	32,8	31,3
27,5	30,4	28,8
27,9	29,7	28,9
25,3	28,9	26,9
31,4	32,6	32
27,9	29,3	29,1
29,1	31,1	30
29,3	30	29,7
29,9	32,3	31,2
26,6	28,9	29,6
29,7	33,4	31,4
29,4	31,5	30,6
29,1	31,5	30,2
28	31,5	29,8
30,7	32,1	31,1
29,1	30,3	29,5
25,7	28,7	26,6
26,9	29,1	28,1

temperatura gemelo min	temperatura gemelo maximo derecho (	temperatura gemelo media de
26,9	31,6	30,8
30,9	32,4	31,3
28,5	31,3	29,5
29,9	32,2	30,9
28,9	30,9	31,3
27,8	29,9	29,3
25,9	28,6	26,8
30	32,2	31,6
27,8	29,7	29,3
28,2	31,3	29,8
28,4	29,4	29
30,9	31,4	31
28,4	31,3	29,8
29,9	32,9	31,9
29,9	31,6	30,9
28,9	30,9	29,9
27,8	30	28,6
29,9	31,5	30,5
28,5	29,8	29,3
25,8	28,5	27
25,7	31,2	28,3

temperatura tendon aqu	temperatura tendon aquiles n	temperatura t	temperatura t	temperatura t
19	29,6	24,8	20,5	30,8
20,1	31,8	27,2	20,6	31,7
20,8	29,7	26,3	20,1	30
25,4	32,4	30,9	23,9	32
24	30,4	29,9	24,1	31,1
25,9	31,8	29,7	25,1	31,9
22,9	28,3	26,1	23,9	27,7
25,3	31,6	29,1	25,9	31,7
22,9	27,9	25,9	23,1	28,1
25,9	29,6	27,9	24,8	29,8
23,9	29	27,9	25,1	30,7
27,1	31,3	29,8	27,3	32,1
27,9	33,3	30,2	29,3	33,4
27,9	31,9	29,8	27,8	33,2
29,9	33,8	31,9	27,5	31,9
26,5	31,9	32,6	28,9	31,8
25,5	29,7	28	24,3	28,6
25,6	31,5	30,5	25,8	31,5
23,9	29,9	28,5	24,9	29,9
26,2	29,4	28,5	26,4	28,8
25,4	32,4	29,9	27,8	31,3

temperatura t	Diferencia des	Diferencia des	Diferencia des	Diferencia des	Diferencia des	Diferencia des
27	3,8	-0,3	-1,2	3,3	-2,4	-1,6
27,7	2	-0,1	0,5	0,8	0,3	0,9
26	1	-1	-0,9	-2,3	-0,2	-0,5
30,1	-0,4	1,1	0,6	0,9	2,6	1,9
29,8	0,4	0	-0,3	0,4	-0,2	-0,3
28,5	-1,5	-1,1	-2,3	-0,8	-2,4	-2
26,3	-1,8	-1,8	-2,1	-0,3	-0,4	-0,7
29,8	-0,8	1	0,4	0,6	0,3	0,3
25,7	0,9	-1,3	-1,2	-1,3	-1,1	-1,2
28,2	0,9	0,1	-0,2	-1,5	-0,8	-0,9
28,7	-3,1	-3	-2,7	-1,1	0	0,2
31,7	-2,5	-2,5	-2,9	-1,5	-1,2	-1,3
30,7	-5,3	-4,5	-4,9	-7	-4,5	-6,3
31,1	0	0,5	0,1	0,2	0,9	1,3
30,1	-7,5	-4,7	-6	-3	-1,5	-1,9
31,8	0	-1	-1,4	-1	-0,4	-0,9
27,3	-5,1	-2,5	-4,2	-3,9	-2,7	-3,6
29,7	1,4	0,9	0,8	-0,2	0,8	0,5
28,8	0	1,3	0,6	-0,3	1,1	0,5
28,1	-1,4	-2,3	-3,1	-4,9	-2,6	-3,8
29,5	0,7	1,3	2,1	1	0,3	0,3

Diferencia des	Diferencia des	Diferencia des	Diferencia des	Diferencia des	Diferencia des	Diferencia des
2,2	-0,6	0,2	1	-1,2	0,2	2,3
3,3	0,8	0,8	1,4	1,3	0,9	0,6
-0,4	1,4	0	0	1,2	0,4	0,1
0	0,3	0,4	0,2	-0,2	-0,3	2,8
-1,4	-0,1	-0,8	0,4	0,3	1,7	2,3
-0,7	-1,2	-1,1	-2,3	-1,2	-1,4	1,6
-0,6	-0,3	-0,8	-0,8	-0,4	-1	1,2
5,5	0,9	1,4	2,1	0,9	1,3	2,7
-1,9	-1,9	-1,5	-1,7	-1,3	-0,9	1,7
0	-0,1	0,2	-0,6	1,1	0,2	2,4
-0,5	-0,6	-0,6	-1,1	-1,3	-1,3	0,7
-0,9	0,6	-0,2	0,4	-0,8	-0,7	2,6
-0,8	-2,9	-0,7	-1,1	-0,5	-0,5	2,5
0,8	2,9	1,3	0	2,2	1,7	3,7
-0,4	0,5	0	0,6	0,6	0,8	-0,3
-0,2	0,3	-0,4	-0,5	-0,1	-0,6	1,8
-2,3	-0,8	-1,8	-2,6	-2,1	-2,8	0,2
1,3	1,1	1,1	1,6	1,2	1,1	3,1
2,3	0,8	1,2	0,6	0,2	0,8	2,3
-4,2	-2,6	-3,7	-3,5	-4	-3,7	5,7
0,2	1,1	0,9	1,9	1,2	0,4	2

Diferencia des	Diferencia des	Diferencia des	Diferencia des	Diferencia despues-antes med aquiles dere
-1,6	-0,9	0,8	-0,7	0,7
3,3	1,8	0,9	1,2	2,4
0,5	0,4	-2	1,3	0,4
1,8	4,3	2	3,5	2,8
1,9	3,7	1,8	2,2	3,3
0,9	0,9	2,6	1,2	0,6
0,5	1,7	1,4	1,7	1,3
3,3	2,3	2,1	2,4	2,5
1	1,6	1,4	1	1,8
2,4	2,1	1,3	1,7	2,1
0,8	0,9	3	2,1	2,5
1,1	1,2	4,2	1,2	2,7
2,2	1,5	2,3	1,9	0,8
2,8	2,2	2,7	1,9	2,3
2,6	1,2	5,2	3,4	4,9
2,3	4,5	4	1,7	0,5
-1,9	-0,1	-1,4	-2,6	-0,7
1,7	4,2	4,4	2	3,6
4,8	4,6	2,9	1,5	4,5
-0,2	2,7	4,4	-1,2	2,1
2,9	3,6	2,4	0,1	1,7

echo
